# Comparative evaluation of diode laser versus argon laser photocoagulation in patients with central serous retinopathy: A pilot, randomized controlled trial [ISRCTN84128484]

**DOI:** 10.1186/1471-2415-4-15

**Published:** 2004-10-29

**Authors:** Lalit Verma, Rajesh Sinha, Pradeep Venkatesh, HK Tewari

**Affiliations:** 1 Dr. Rajendra Prasad Centre for Ophthalmic Sciences, All India Institute of Medical Sciences, New Delhi, India

**Keywords:** diode laser, central serous retinopathy, laser in CSR

## Abstract

**Background:**

To evaluate the efficacy of diode laser photocoagulation in patients with central serous retinopathy (CSR) and to compare it with the effects of argon green laser.

**Methods:**

Thirty patients with type 1 unilateral CSR were enrolled and evaluated on parameters like best corrected visual acuity (BCVA), direct and indirect ophthalmoscopy, amsler grid for recording scotoma and metamorphopsia, contrast sensitivity using Cambridge low contrast gratings and fluorescein angiography to determine the site of leakage.

Patients were randomly assigned into 2 groups according to the statistical random table using sequence generation. In Group 1 (n = 15), diode laser (810 nm) photocoagulation was performed at the site of leakage while in Group 2 (n = 15), eyes were treated with argon green laser (514 nm) using the same laser parameters. Patients were followed up at 4, 8 and 12 weeks after laser.

**Results:**

The mean BCVA in group 1 improved from a pre-laser decimal value of 0.29 ± 0.14 to 0.84 ± 0.23 at 4 weeks and 1.06 ± 0.09 at 12 weeks following laser. In group 2, the same improved from 0.32 ± 0.16 to 0.67 ± 0.18 at 4 weeks and 0.98 ± 0.14 at 12 weeks following laser. The improvement in BCVA was significantly better in group 1 (p < 0.0001) at 4 weeks. At 4 weeks following laser, all the patients in group1 were free of scotoma while 6 patients in group 2 had residual scotoma (p < 0.05). The mean contrast sensitivity in group 1 improved from pre-laser value of 98.4 ± 24.77 to 231.33 ± 48.97 at 4 weeks and 306.00 ± 46.57 at 12 weeks following laser. In group 2, the same improved from 130.66 ± 31.95 to 190.66 ± 23.44 at 4 weeks and 215.33 ± 23.25 at 12 weeks. On comparative evaluation, a significantly better (p < 0.001) improvement was noted in group 1.

**Conclusion:**

Diode laser may be a better alternative to argon green laser whenever laser treatment becomes indicated in patients with central serous retinopathy in terms of faster visual rehabilitation and better contrast sensitivity. In addition, diode laser also has the well-recognized ergonomic and economic advantages.

## Background

Central serous retinopathy (CSR) is a retinal disorder affecting young adults, characterized clinically by a well-defined, translucent, circumscribed detachment of neurosensory retina at the posterior pole, usually involving the macula. The detachment results from accumulation of transparent fluid in the potential space between retinal pigment epithelial layer and the neurosensory retina. However, the source of this fluid and the exact etiopathogenesis of this disorder remain ill understood. Maumenee [1] described the concept of leakage site within the retinal pigment epithelial layer following fluorescein angiographic studies in patients with central serous retinopathy. Recent studies [2-4], using indocyanine green angiography, have indicated that the possible site of primary pathology in central serous retinopathy is the choroidal vessels and the involvement of the pigment epithelial layer is only secondary to it.

Although, other treatment modalities have been suggested for central serous retinopathy, argon green (514 nm) laser photocoagulation has been the most widely accepted modality. The 810 nm-diode laser owing to deeper penetration should prove to be more efficacious in the treatment of central serous retinopathy given the current thinking that CSR may primarily be a choroidal disease. The present study was undertaken to test the efficacy of diode laser photocoagulation in central serous retinopathy in comparison to argon green laser. According to the best of our knowledge, this is the first clinical study in which a comparative evaluation between the above two groups has been done in central serous retinopathy.

## Methods

Thirty patients of unilateral central serous retinopathy presenting to the retina services at the Rajendra Prasad Centre for Ophthalmic Sciences were selected for the study. They were randomly assigned into 2 groups according to the statistical random table using sequence generation. As it was a pilot study we decided only the convenient sample size i.e. 15 in each group. The allocation of patients into 2 groups was done by a person who was not involved in the study and the sequence was concealed until interventions were assigned to prevent bias. The patient selection was based on the following inclusion criteria: age less than 50 years, type 1 CSR with a single leak on fluorescein angiography that was at least 300 microns away from fovea, presence of an indication for laser treatment (recurrence, occupational, history of poor visual outcome in fellow eye) and no history of any treatment in the past. Patients with multiple leak CSR, type 2 or type 3 CSR or leak at papillomacular bundle or leak within 300 μ from the foveal centre were not included in the study. Patients with other ocular pathology such as choroidal neovascularization, choroidal inflammatory or neoplastic disorder or a congenital optic nerve pit were also excluded from the study.

Informed consent was obtained from all the patients included in the study before initiating treatment and an approval of the institute body ethics committee was obtained to undertake the study. In all the patients the following parameters were evaluated before laser treatment and also during the follow-up period: best corrected visual acuity (BCVA) on Snellen's acuity chart, fundus examination by direct and indirect ophthalmoscopy, amsler grid for determining the degree of scotoma and / or metamorphopsia, contrast sensitivity using Cambridge low contrast gratings and fluorescein angiography (Canon CF-60 UD camera using 400ASA, 35-mm black and white film).

Technique of scoring scotoma and metamorphopsia on the amsler grid is described below:

The amsler grid consists of a 10-cm square divided into smaller 5 mm squares. When it is viewed at one-third of a meter, each small square subtends an angle of 1°. The patient was asked to look directly at the central spot with the uncovered eye and to mark out any black spot, distortion of lines, or blurred area on the grid. The number of small squares within the area delineated by the patient was counted and scotoma and metamorphopsia score was graded numerically as shown in table 1 and 2.

Patients included in the study were randomly assigned to one of the following two groups. In group 1 (n = 15), the site of leakage was treated by diode laser (810 nm). Laser was performed with Volk area centralis contact lens (Keeler Ltd, Clewer Hill Road, Windsor, Berks, SL4 4AA) and the parameters used were spot size of 100 microns, duration of 100 to 200 milliseconds and power just enough to produce minimal greying. In group 2 (n = 15), patients were treated with argon green laser (514 nm) following the same laser parameters. Patients were followed up at intervals of 4, 8 and 12 weeks after laser treatment and the results were analyzed, which included a comparative analysis of the outcome between the two groups. Descriptive statistics i.e. mean and standard deviation was calculated for each variable at each follow-up for the 2 groups. To see the significant difference between the 2 groups at each point we applied "student 't' test" (unpaired). To see the trend within the group we applied "two way analysis of variance (ANOVA)". 'P' value of 0.05 was considered as statistically significant. BMDP 7.0 statistical package was used for the statistical analysis.

## Results

The average age of the patients in group 1 was 34.06 ± 2.54 years (range: 30–40 years) and in group 2, it was 34.66 ± 3.23 years (range: 30–42 years). Twelve cases (80%) in group 1 and 13 patients (86.66%) in group 2 were males. All the patients presented to us within eight days (range: 3–8 days) of onset of visual symptoms. The average number of laser spots applied to the leakage site in group 1 was 3.13 ± 0.71 (range: 2–4) and in group 2 it was 3.26 ± 0.77 (range: 2–4). All the patients came regularly for the follow-up as per the protocol during entire period of study. The study was performed during the period between January 1998 and June 2000.

**Figure 1:**
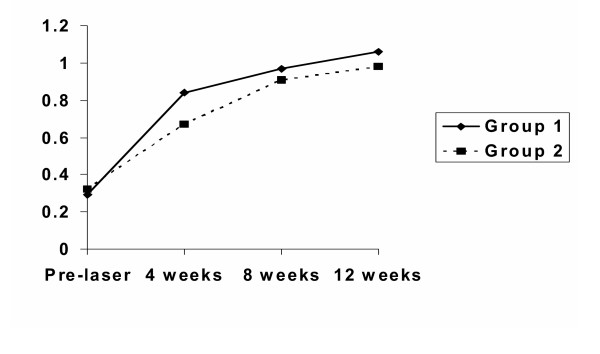
Best Corrected Visual Acuity – Group 1 (Diode) Vs Group 2 (Argon green)

The mean best-corrected visual acuity (BCVA) in decimal at the time of presentation in patients assigned to group 1 was 0.29 ± 0.14. Best corrected visual acuity four weeks after diode laser treatment had improved to 0.84 ± 0.23. The BCVA further improved to 0.97 ± 0.17 and 1.06 ± 0.09 at 8 weeks and 12 weeks respectively. In group 2, the mean BCVA at the time of presentation was 0.32 ± 0.16 and it improved to 0.67 ± 0.18 at 4 weeks follow-up after argon green laser photocoagulation. Further improvement in visual acuity to 0.91 ± 0.19 and 0.98 ± 0.14 at 8 weeks and 12 weeks follow up respectively was observed. Statistical analysis of data pertaining to improvement of BCVA in the two groups by student **'t' **test, showed a statistically significant difference at 4 weeks with patients treated by diode laser (group 1) having a better improvement (p < 0.001) (Figure 1). This difference decreased and was not found to be statistically significant during later follow-up at 8 and 12 weeks.

All the patients in both the groups had central scotoma at the time of presentation. At the first follow up at 4 weeks following laser treatment, all the patients in group 1 (n = 15) were free of scotoma while 6 patients in group 2 (n = 15) had residual scotoma of grade 1 (involving 1–50 small squares on the amsler grid). On comparative evaluation, this difference was found to be statistically significant (p < 0.05). At 8 weeks and 12 weeks follow up, none of the patients had residual scotoma; however, 3 patients in group 2 developed a paracentral scotoma corresponding to the site of laser scar.

Four patients in group 1 and five in group 2 had complaints of metamorphopsia at the time of presentation. At the first follow up at 4 weeks, 2 cases in group 1 and 4 cases in group 2 were still having grade 1 metamorphopsia. On comparative evaluation, this difference in the two groups was not found to be significant statistically. At 8 weeks and 12 weeks follow up, all patients in both the groups were free of metamorphopsia.

**Figure 2:**
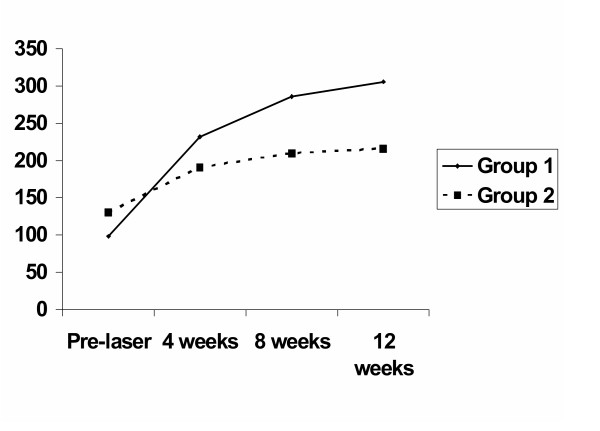
Contrast Sensitivity – Group 1 (Diode) Vs Group 2 (Argon green)

Contrast sensitivity was evaluated using the Cambridge low contrast gratings. The reference normal for our patients as observed in earlier studies at our centre has been 340 (range 310–370) (unpublished data). The mean absolute value of contrast sensitivity at the time of presentation in group 1 was 98.4 ± 24.77. This improved to 231.33 ± 48.97 at the first follow up at 4 weeks following diode laser photocoagulation. It further improved to 286.00 ± 48.52 at 8 weeks follow up and 306.00 ± 46.57 at the third follow up at 12 weeks. In group 2, the mean value of contrast sensitivity at the time of presentation was 130.66 ± 31.95, which improved to 190.66 ± 23.44 at 4 weeks following argon green laser treatment. This value further improved to 210.00 ± 27.25 at 8 weeks and 215.33 ± 23.25 at 12 weeks follow up. Comparative evaluation of the improvement in contrast sensitivity by student **'t' **test between the two groups showed that there was an initial rapid improvement in contrast sensitivity in both the groups but group I patients had a significantly greater improvement than group 2 patients at all the follow up visits (p < 0.001) (Figure 2).

In both the groups, the commonest site of fluorescein leakage was the area superonasal to fovea. Fluorescein angiography repeated 4 weeks later showed that in both the groups, all the patients had complete resolution of the leakage site. No recurrence was reported till completion of follow up at 12 weeks.

## Discussion

Visual prognosis in central serous retinopathy in most cases is good as they resolve spontaneously. Klein M et al. [5] studied and followed up 34 eyes of CSR without giving any treatment and found that final visual acuity was 20/30 or better. The average time for the fluid to resolve was between 3 months to 6 months and there was a recurrence rate of 45%. Sequelae such as macular degeneration and chronic retinal pigment epithelial decompensation are rare after the first episode. However, in a small group of patients visual prognosis has been reported to be less favorable [6,7].

Encouraging results of laser photocoagulation has been found in various studies which conclude that laser photocoagulation could effectively decrease the time for visual recovery in patients with central serous retinopathy [8-10]. However, the ultimate visual acuity despite laser photocoagulation remained no different from patients in whom no treatment was done and that recurrences could not be prevented by laser photocoagulation [10].

Results of treatment of the leakage site in patients with central serous retinopathy with ruby [9], krypton [11,12] and argon lasers [9,10,13,14] and Xenon arc [15] have been reported in the literature. Although, Slusher [11] suggested that the wavelength emitted by krypton laser might be more tissue-selective, argon green laser photocoagulation has been the most widely accepted treatment modality in patients with central serous retinopathy [9,10,12-14].

Studies performed with indocyanine green angiography in cases of central serous retinopathy have reported that hyperpermeability of choroidal vessels could be the primary pathology in CSR and retinal pigment epithelial abnormality is only secondary to it [2-4].

Brancato R et al [16] performed transpupillary chorioretinal photocoagulation in rabbit eyes using a diode laser prototype and a commercially available argon laser and found that they were similar on ophthalmoscopy. However, histopathological examination performed twenty-four hours after the treatment showed that argon irradiation resulted in damage to both the inner and outer retinal layers while the diode laser radiation produced damage to outer retina and choroid. If this were true, energy delivered by argon laser (514 nm) seems inadequate in being able to reach the deeper choroidal layers and treat the primary site of pathology. This inherent limitation of argon laser in view of recent ICG reports on the pathogenesis of central serous retinopathy could also explain the less than ideal outcome in the earlier studies using this laser in the treatment of patients with central serous retinopathy. The 810 nm-diode laser emission is in the near infrared (810 nm) region and this wavelength is able to reach the deeper choroidal layers [16-18]. Hence, we felt that the 810 nm-diode laser may theoretically be more efficacious in the laser treatment of central serous retinopathy.

Our observations showed that following diode laser treatment in patients with central serous retinopathy, the recovery of visual acuity was more rapid in comparison to the argon laser treatment group. Further, all the patients treated with diode laser showed complete resolution of scotoma at the first follow up at 4 weeks and none of them developed a scotoma corresponding to the site of laser treatment. In contrast, in patients subjected to argon green laser treatment, there was a significant residual scotoma at 4 weeks and 20% of the patients developed scotoma corresponding to the laser scar.

Following laser treatment, contrast sensitivity in both the groups improved rapidly in the first four weeks. However, there was significantly greater improvement in contrast sensitivity in eyes subjected to diode laser. This observation is indicative that diode laser is more beneficial with regard to conservation of contrast sensitivity following laser treatment in patients with central serous retinopathy.

Development of subretinal neovascularization following laser treatment for central serous retinopathy has been described earlier in literature [19]. However, in our study none of the patients in any group developed this complication.

The present study suggests that diode laser photocoagulation brings about a faster quantitative and qualitative visual recovery in comparison to argon green laser in eyes with central serous retinopathy. The main aim of performing laser photocoagulation in eyes with CSR is to hasten resolution and this is better fulfilled by diode laser. Further, as argon laser can hit up to the outer retinal layers, it can seal the site of leaking retinal pigment epithelium, but it cannot reduce the fluid overload over the pigment epithelial layer caused by hyperpermeability of choriocapillaries, which can be the cause of recurrence of CSR. As diode laser hits the choroid, which is the exact site of pathology, it should reduce the fluid overload on the retinal pigment epithelium.

## Conclusions

In the present study, our observations are suggestive that diode laser may be a better alternative to argon laser whenever laser treatment is indicated in patients with central serous retinopathy. In addition, diode laser also has the well-recognized economic and ergonomic advantages of being less costly, more efficient, portable and easy to maintain [20,21], which is also a matter of concern particularly in third world countries.

## Declaration of competing interest

The author(s) declare that they have no competing interests.

## Authors' contributions

LV designed the study and performed laser. RS performed the data collection and wrote the manuscript. PV followed up the patients. HKT performed the angiography.

All authors read and approved the final manuscript.

Presented in part in the Annual Meeting of the American Academy of Ophthalmology, 1997 in San Francisco, California.

## Pre-publication history

The pre-publication history for this paper can be accessed here:



## Figures and Tables

**Table 1:** Amsler Grid: Scotoma Score

**No of small squares**	**Grade**
0	0
1 – 50	I
51 – 100	II
101 – 150	III
151 – 200	IV

**Table 2:** Amsler Grid: Metamorphopsia Score

**No of small squares**	**Grade**
0	0
1 – 25	I
26 – 50	II
51 – 75	III
76 – 100	IV
